# Willingness to bear economic costs of measures against SARS-CoV-2 in Germany

**DOI:** 10.1186/s12889-021-11734-4

**Published:** 2021-09-17

**Authors:** Hans-Helmut König, Freia De Bock, Philipp Sprengholz, Benedikt Kretzler, André Hajek

**Affiliations:** 1 Department of Health Economics and Health Services Research, University Medical Center Hamburg-Eppendorf, Hamburg Center for Health Economics, Martinistraße 52, 20246 Hamburg, Germany; 2 Federal Centre for Health Education, Cologne, Germany; 3 Department of Health Communication, University of Erfurt, Erfurt, Germany

**Keywords:** Willingness to accept, Willingness to pay, Covid-19, SARS-CoV-2, Corona-virus, Economic costs, Income

## Abstract

**Background:**

The aim of this study was to assess the willingness of the general population in Germany to bear the economic costs of measures against the spread of SARS-CoV-2.

**Methods:**

Repeated cross-sectional data were taken from three waves of a nationally representative survey of individuals aged 18 to 74 years (wave 8: 21–22 April 2020, *N* = 976; wave 16: 7–8 July 2020, *N* = 977; wave 38: 9–10 March 2021). The willingness to accept a reduction of annual household income in order to bear the economic costs of the measures against SARS-CoV-2 served as outcome measure. Two-part models were used including explanatory variables on sociodemographic and (subjectively assessed) potential health hazard caused by COVID-19.

**Results:**

65.5% (61.6%; 56.9%) of respondents in wave 8 (wave 16; wave 38) were willing to accept a reduction of income, with the likelihood for accepting a reduction of income being positively associated with higher affect (i.e. emotional reaction) and presumed severity regarding COVID-19 in all three waves. The mean maximum percentage of income participants were willing to give up was 3.3% (95% CI: 2.9 to 3.7%) in wave 8, 2.9% (95% CI: 2.5 to 3.3%) in wave 16 and 4.3% (95% CI: 3.6 to 5.0%) in wave 38, with presumed severity of COVID-19 being positively associated with this percentage in all three waves.

**Conclusions:**

The majority of respondents indicated willingness to sacrifice income in order to bear the costs of measures against the spread of SARS-CoV-2, with the potential health hazard caused by COVID-19 being consistently associated with this willingness. However, the proportion of individuals who were willing to give up income slightly decreased throughout the pandemic.

**Supplementary Information:**

The online version contains supplementary material available at 10.1186/s12889-021-11734-4.

## Introduction

Measures aimed at preventing the spread of SARS-CoV-2 by reducing social contacts, specifically so-called (near) lock-downs, have a substantial negative impact on the economy. For example, the government’s order to temporary close businesses, ban on trade fairs and cultural events, or closure of schools decreases the economy’s productivity. According to the German Federal Statistical Office the gross domestic product (GDP) in Germany fell by 4.8% in 2020 [1], which is similar to the GDP decline of 4.9% in the Organisation for Economic Co-operation and Development (OECD) area, being the largest fall ever recorded since 1962 [2]. Real mean wages in Germany decreased by 1.1% in 2020, mainly due to 2.9% fewer hours worked per week [3]. However, due to stabilizers like short time compensations (so-called “Kurzarbeitergeld”) and economic stimulus packages, private disposable incomes have stayed relatively stable during the COVID-19 pandemic so far. In return, public debt in Germany increased by 273 billion Euro (14.4%) in 2020 [4], which eventually will have to be borne by the citizens.

In Germany, nationwide implementation of measures to prevent the spread of SARS-CoV-2 started on 16 March 2020, including, among others, closings of schools or day-care centers. On 22 March 2020 travel bans and contact restrictions in public were added and repeatedly prolonged during the following weeks (first lock-down). On 20 April 2020, some restrictions were lifted, e.g. shops up to a certain size were allowed to reopen. Schools started to open gradually in early May 2020. In the course of May 2020, further restrictions were lifted such as contact bans or closing of museums or playgrounds. In June 2020, restrictions were cut back further but were to be tightened again locally in case the number of infections increased above a threshold of 50 per 100,000 inhabitants per week. After a sharp increase of the infection rate in autumn 2020, contact restrictions and closure of, e.g., restaurants and theaters were imposed on 2 November 2020, followed by firmer contact restrictions, the closure of schools and of most shops (second lockdown) on 16 December 2020. On 3 March 2021, federal and state governments agreed on a stepwise lifting of restrictions in case the infection rate decreased below 50 per 100,000 inhabitants per week (which applied to only few regions at that time). Starting from 24 April 2021 even stronger restrictions including a nightly curfew were introduced by the federal government in case the infection rate increased over 100 per 100,000 inhabitants per week.

While a large proportion of the German population seems to have agreed with the government’s policy to prevent the spread of SARS-CoV-2, [5] little is known about the population’s willingness to bear the associated economic burden which has not become fully visible yet. Yet, in order to assess the acceptance of this in a longer perspective, it is important to know whether and to what extend the population is willing to accept the economic consequences, e.g., in terms of sacrificing income and wealth. To our knowledge, only one study from Poland has analyzed the willingness to bear the economic costs in the fight against the COVID-19 pandemic so far [6]. However, using a convenience sample and a mixed experimental design, the Polish study focused on the psychological mechanisms (such as fear or feeling of control) that determine people’s willingness to bear the economic costs in terms of unemployment and inflation. Therefore, the current study aimed at assessing the willingness of the German population to bear the economic cost of the measures against the spread of SARS-CoV-2 in terms of willingness to accept a reduction of annual household income. Furthermore, factors that might affect this willingness were explored.

## Materials and methods

### Sample

For this study, repeated cross-sectional data were taken from wave 8, wave 16, and wave 38 of the COVID-19 Snapshot Monitoring (COSMO) [7]. The individuals participating in wave 8 are different from those individuals participating in wave 16 and wave 38, respectively. We restricted our study to these waves since the dependent variable was solely quantified in these three waves. The first wave of the COSMO study took place in early March 2020 (3rd to 4th March 2020; 15 min online questionnaire). Further waves took place every week, with wave 8 taking place from 21st to 22nd April 2020, wave 16 taking place from 7th to 8th July 2020 and wave 38 taking place from 9th to 10th March 2021. German speaking adults aged 18 to 74 years and residing in Germany were included. The market research company “Respondi” recruited the participants (ISO 26362 certified online sample provider). Sampling was quota-based (non-probability quota sample). The individuals were recruited from an online-panel in a way that it reflects the distribution of gender and age (crossed-quota: gender x age) and federal state (uncrossed) in the total German population [8]. On the basis of the quotas, individuals are admitted to the survey or screened out on the first page. A sample size of about *n* = 1000 per wave was used to identify small effects and to ensure representativeness [7].

### Dependent variable

First, participants were introduced to the topic with the following statement: “Economic experts have calculated that the measures against the spread of the corona virus (e.g. closure of businesses, ban on events) will cause considerable economic costs”. In order to elicit the participants’ preferences for these measures, we applied a simple form of the contingent valuation method [9–11] by asking the participants for their maximum willingness-to-pay (in terms of giving up income) to bear the economic costs of these measures. More precisely, participants were asked to rate the statement: “What is the maximum percentage of your annual household income that you would be willing to give up in order to bear the economic costs of the measures against the corona virus?” (answer categories: 0%; 1–2%; 3–5%; 6–10%; 11–25%; 26–50%; more than 50%). The high face validity of our dependent variable was affirmed by a pretest with *n* = 15 individuals.

### Independent variables

Based on theoretical considerations and empirical studies, we used various classical sociodemographic variables describing living situation and social stratum as well as variables indicating the probability of infection and the (subjectively assessed) potential health hazard caused by COVID-19 as explanatory variables. Sociodemographic variables included age group (four categories: 18 to 29 years; 30 to 49 years; 50 to 64 years; ≥ 65 years), gender (women; men), marriage/relationship (no; yes), living arrangement (living alone; two or more individuals in the same household), presence of children under 18 years in household (no; yes), educational level (up to 9 years/10 and more years (excluding general qualification for university entrance); 10 years and more (including general qualification for university entrance)), self-employment (no; yes), background of migration (no; yes), region (state of former West Germany; state of former East Germany) and size of the town (four categories: municipality/small town (1–20,000 inhabitants); medium sized town (20,001–100,000 inhabitants); small city (100,001–500,000 inhabitants); big city (> 500,000 inhabitants). The prevalence of COVID-19 in the region (COVID-19 cases per 100,000 population (below median; above median)) was used as an indicator of the probability of infection. The presence of chronic conditions (no; yes) was used as an indicator for morbidity which might increase the potential health hazard caused by COVID-19. Furthermore, the subjectively presumed severity of the personal potential health hazard caused by COVID-19 was used as explanatory variable (7-point scale; higher values reflect higher presumed severity: “How would you rate an infection with the novel corona virus for yourself?” (from 1 = completely harmless to 7 = extremely dangerous)). Lastly, subjective affect (i.e. emotional reaction) regarding COVID-19 was included as explanatory variable. This scale consists of seven items. For instance, it was asked: “For me, the new type of corona virus is ... ‘near’ ‘scary’ (1) to ‘far away’ ‘not scary’ (7)”. The final score was calculated by averaging the items. In our study, Cronbach’s alpha equaled .78 (wave 8), .80 (wave 16), and .77 (wave 38).

In a first robustness check (wave 8 and wave 16 for reasons of data availability), it was additionally adjusted for personal COVID-19 infection (no; don’t know; yes [including: yes, confirmed; yes, but unconfirmed; yes, recovered) and COVID-19 infection in personal environment (no; don’t know; yes [including: there are unconfirmed cases; there are confirmed cases; there are individuals who have recovered; there are deceased individuals]).

In a second robustness check (wave 16 and wave 38 for reasons of data availability), it was additionally adjusted for household net income prior to the COVID-19 pandemic [categories: lower than 1250 Euro; 1250 Euro to lower than 1750 Euro; 1750 Euro to lower than 2250 Euro; 2250 Euro to lower than 3000 Euro; 3000 Euro to lower than 4000 Euro; 4000 Euro to lower than 5000 Euro; 5000 Euro and above].

### Statistical analysis

Sample characteristics (wave 8, wave 16 and wave 38) are calculated in total as well as stratified by the willingness to accept any reduction of annual household income (no (0%) vs. yes (> 0%). To test differences between these two groups, t-tests or Chi^2^-tests were used, as appropriate. Moreover, a figure was used to show the distribution of maximum accepted income reduction (wave 8, wave 16, and wave 38).

Besides applying descriptive statistics, we analyzed the willingness to accept a reduction of annual household income (in %) by using Two Part Models [12] (first part: logit model; second part: generalized linear model with gamma distribution and log link function taking into account the highly skewed distribution of positive values [13] – supported by AIC and BIC values). Categorical data on the percentage of income were set at the midpoint of the respective category’s interval to obtain metric data (e.g., 0, 1.5, 4%, and so on). Two Part Models are commonly used in cost analysis when there is a large amount of zeros (in our case, no willingness to accept reduction of income) [14]. Thus, we also used them in our current study. The “twopm” command in Stata was used [12]. Average marginal effects were computed and displayed. They offer the advantage of interpretability, which means that they reflect the change in the outcome measure (willingness to accept a reduction of income in %) associated with a one-unit change of the explanatory variable (for continuous variables; for categorical variables: the difference to the reference category). The level of significance was fixed at 5%.

In a third robustness check, Two Part models were replaced by linear regressions. In a fourth robustness check, interval regressions were used.

### Ethics

The study was approved by the institutional review board at the University of Erfurt (#20200501). Participants provided informed consent prior to entering the study (at all waves).

## Results

### Sample characteristics

**Fig. 1:**
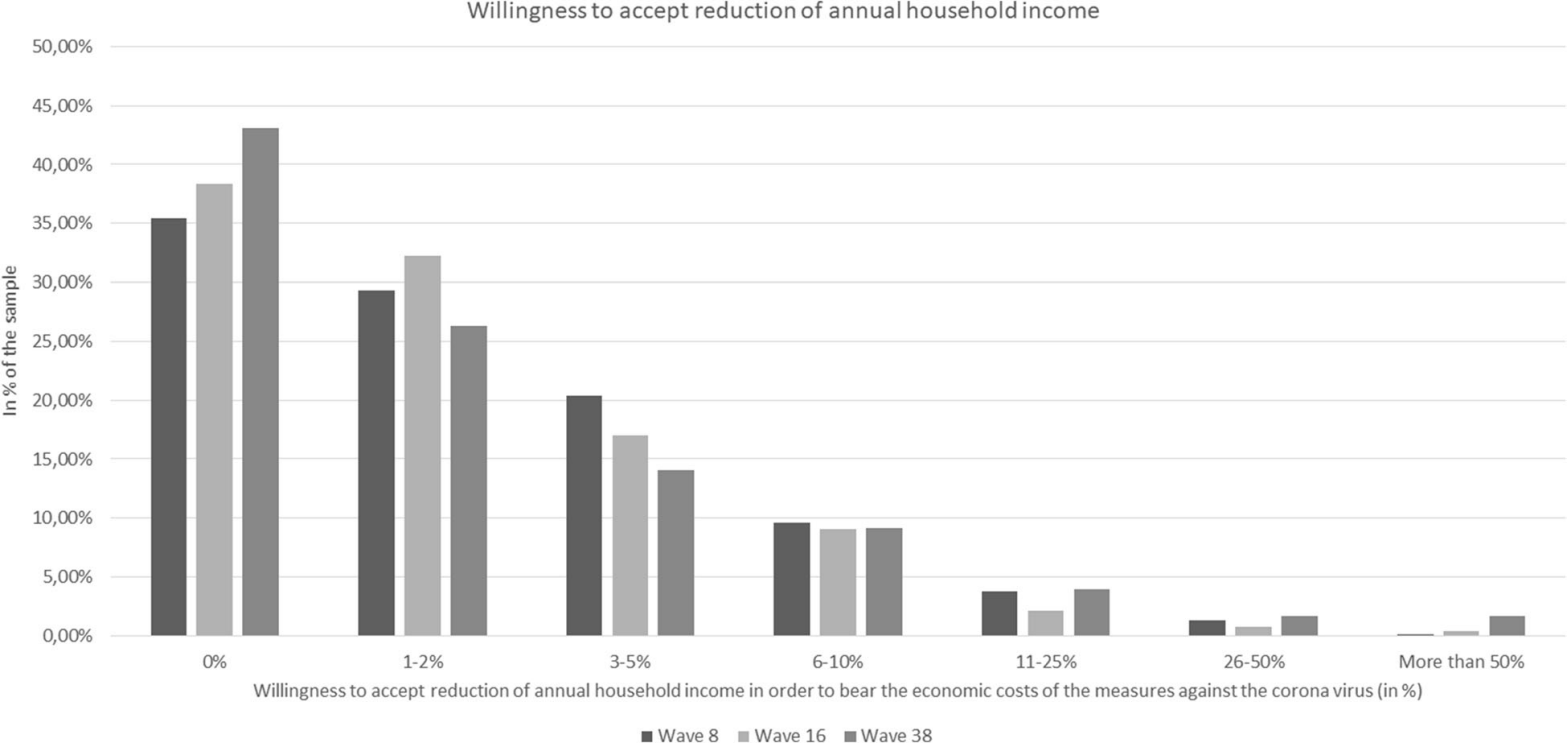
Percentage of sample willing to accept reduction of annual household income in order to bear the economic costs of the measures against the corona virus

In Table 1, sample characteristics for wave 8 (*n* = 976), wave 16 (*n* = 977) and wave 38 (*n* = 958) are displayed in total, and stratified by the willingness to accept any reduction of annual household income (no (0%) vs. yes (> 0%)). Sociodemographic characteristics were similar in all three waves, with mean age being 47.0 years (SD: 15.9) in wave 8, 48.0 years (SD: 15.3) in wave 16 and 45.4 years (SD: 15.3 years) in wave 38, ranging from 18 to 74 years in all three waves. The proportion of individuals who were not willing to accept any reduction of income was 35.5% in wave 8, 38.4% in wave 16 and 43.1% in wave 38. The mean maximum percentage of income participants were willing to give up was 3.3% (95% CI: 2.9 to 3.7%) in wave 8, 2.9% (95% CI: 2.5 to 3.3%) in wave 16 and 4.3% (95% CI: 3.6 to 5.0%) in wave 38. The proportion of individuals not willing to accept any reduction of income was significantly higher in wave 38 compared to wave 8 (*p* < 0.001) and wave 16 (*p* = 0.04) whereas the percentage of income participants were willing to give up was significantly higher in wave 38 compared to wave 8 (*p* = 0.01) and wave 16 (p < 0.001). Figure 1 illustrates the distribution of maximum accepted income reduction for all three waves. A reduction of more than 5% (10%) was accepted by only 14.8% (5.2%) of participants in wave 8, 12.4% (3.3%) of participants in wave 16, and 16.5% (7.3%) of participants in wave 38. Stratified analyses showed that in all three waves the willingness to accept any reduction of annual household income (yes (> 0%) vs. no (0%)) was significantly positively associated higher education, living in a state of former West Germany, higher affect regarding COVID-19 and higher presumed severity of COVID-19. Further significant positive associations could be found with the number of COVID-19 cases per 100,000 population in wave 8, with male gender, household size and the absence of chronic diseases in wave 16, and with age category as well as migration background in wave 38.

**Table 1:** Sample characteristics for the analytical sample

	Wave 8 (2 days after end of first lockdown, April 21–22, 2020)				Wave 16 (No lockdown, few restrictions, July 7–8, 2020)				Wave 38 (Second lockdown, March 9–10, 2021)			
	Total sample	Individuals with WTA = 0% (*n* = 346 individuals)	Individuals with WTA > 0% (*n* = 630 individuals)	*p*-value	Total sample	Individuals with WTA = 0% (*n* = 375 individuals)	Individuals with WTA > 0% (*n* = 602 individuals)	*p*-value	Total sample	Individuals with WTA = 0% (*n* = 413 individuals)	Individuals with WTA > 0% (*n* = 545 individuals)	*p*-value
	Mean (SD)/n (%)	Mean (SD)/n (%)	Mean (SD)/n (%)		Mean (SD)/n (%)	Mean (SD) / n (%)	Mean (SD) / n (%)					
Sex:				.20				.01				.20
Men	470 (48.2%)	153 (45.4%)	313 (49.7%)		479 (49.0%)	165 (44.0%)	314 (52.2%)		475 (49.6%)	195 (47.2%)	280 (51.4%)	
Women	506 (51.8%)	189 (54.6%)	317 (50.3%)		498 (51.0%)	210 (56.0%)	288 (47.8%)		483 (50.4%)	218 (52.8%)	265 (48.6%)	
Age category:				.34				.09				.01
18 to 29 years	159 (16.3%)	48 (13.9%)	111 (17.6%)		178 (18.2%)	56 (14.9%)	122 (20.3%)		172 (18.0%)	55 (13.3%)	117 (21.5%)	
30 to 49 years	378 (38.7%)	136 (39.3%)	242 (38.4%)		370 (37.9%)	148 (39.5%)	222 (36.9%)		371 (38.7%)	163 (39.5%)	208 (38.2%)	
50 to 64 years	289 (29.6%)	102 (29.5%)	187 (29.7%)		271 (27.7%)	115 (30.7%)	156 (25.9%)		273 (28.5%)	127 (30.7%)	146 (26.8%)	
65 years and over	150 (15.4%)	60 (17.3%)	90 (14.3%)		158 (16.2%)	56 (14.9%)	102 (16.9%)		142 (14.8%)	68 (16.5%)	74 (13.6%)	
Children under 18 years:				.63				.85				.40
No	716 (73.4%)	257 (74.3%)	459 (72.9%)		751 (76.9%)	287 (76.5%)	464 (77.1%)		645 (67.3%)	272 (65.9%)	373 (68.4%)	
Yes	260 (26.6%)	89 (25.7%)	171 (27.1%)		226 (23.1%)	88 (23.5%)	138 (22.9%)		313 (32.7%)	141 (34.1%)	172 (31.6%)	
Education:				<.01				<.001				<.01
up to 9 years / 10 years and more (without general qualification for university entrance)	428 (43.9%)	172 (49.7%)	256 (40.6%)		456 (46.7%)	205 (54.7%)	251 (41.7%)		417 (43.5%)	200 (48.4%)	217 (39.8%)	
10 years and more (with general qualification for university entrance)	548 (56.1%)	174 (50.3%)	374 (59.4%)		521 (53.3%)	170 (45.3%)	351 (58.3%)		541 (56.5%)	213 (51.6%)	328 (60.2%)	
Town size:				.54				.56				.09
Municipality/small town (1–20,000)	369 (37.8%)	135 (39.0%)	234 (37.1%)		362 (37.1%)	146 (38.9%)	216 (35.9%)		357 (37.3%)	170 (41.2%)	187 (34.3%)	
Medium sized town (20,001 – 100,000)	230 (23.6%)	84 (24.3%)	146 (23.2%)		254 (26.0%)	94 (25.1%)	160 (26.6%)		258 (26.9%)	111 (26.9%)	147 (27.0%)	
Small city (100,001 – 500,000)	176 (18.0%)	54 (15.6%)	122 (19.4%)		174 (17.8%)	70 (18.7%)	104 (17.3%)		165 (17.2%)	60 (14.5%)	105 (19.3%)	
Big city (> 500,000)	201 (20.6%)	73 (21.1%)	128 (20.3%)		187 (19.1%)	65 (17.3%)	122 (20.2%)		178 (18.6%)	72 (17.4%)	106 (19.4%)	
Region:				<.01				<.01				<.01
West Germany	833 (85.3%)	281 (81.2%)	552 (87.6%)		819 (83.8%)	299 (79.7%)	520 (86.4%)		797 (83.2%)	328 (79.4%)	469 (86.1%)	
East Germany	143 (14.7%)	65 (18.8%)	78 (12.4%)		158 (16.2%)	76 (20.3%)	82 (13.6%)		161 (16.8%)	85 (20.6%)	76 (13.9%)	
Cases/100,000 population:				<.001				.24				.54
Below median	450 (46.1%)	186 (53.8%)	264 (41.9%)		446 (45.7%)	180 (48.0%)	266 (44.2%)		430 (44.9%)	190 (46.0%)	240 (44.0%)	
Above median	526 (53.9%)	160 (46.2%)	366 (58.1%)		531 (54.3%)	195 (52.0%)	336 (55.8%)		528 (55.1%)	223 (54.0%)	305 (56.0%)	
Relationship/Marriage:				.34				.22				.44
No	300 (30.7%)	113 (32.7%)	187 (29.7%)		334 (34.2%)	137 (36.5%)	197 (32.7%)		289 (30.2%)	130 (31.5%)	159 (29.2%)	
Yes	676 (69.3%)	233 (67.3%)	443 (70.3%)		643 (65.8%)	238 (63.5%)	405 (67.3%)		669 (69.8%)	283 (68.5%)	386 (70.8%)	
Living situation:				.08				<.01				.26
Living alone	244 (25.0%)	98 (28.3%)	146 (23.2%)		251 (25.7%)	116 (30.9%)	135 (22.4%)		231 (24.1%)	107 (25.9%)	124 (22.8%)	
At least 2 individuals in the same household	732 (75.0%)	248 (71.7%)	484 (76.8%)		726 (74.3%)	259 (69.1%)	467 (77.6%)		727 (75.9%)	306 (74.1%)	421 (77.2%)	
Migration background:				.46				.59				<.01
No	840 (86.1%)	294 (85.0%)	546 (86.7%)		842 (86.2%)	326 (86.9%)	516 (85.7%)		794 (82.9%)	358 (86.7%)	436 (80.0%)	
Yes	136 (13.9%)	52 (15.0%)	84 (13.3%)		135 (13.8%)	49 (13.1%)	86 (14.3%)		164 (17.1%)	55 (13.3%)	109 (20.0%)	
Self-employment:				.12				.32				.12
No	880 (90.2%)	305 (88.1%)	575 (91.3%)		893 (91.4%)	347 (92.5%)	546 (90.7%)		875 (91.3%)	384 (93.0%)	491 (90.1%)	
Yes	96 (9.8%)	41 (11.9%)	55 (8.7%)		84 (8.6%)	28 (7.5%)	56 (9.3%)		83 (8.7%)	29 (7.0%)	54 (9.9%)	
Chronic disease:				.72				<.01				.05
No	633 (64.9%)	227 (65.6%)	406 (64.4%)		604 (61.8%)	212 (56.5%)	392 (65.1%)		602 (62.8%)	245 (59.3%)	357 (65.5%)	
Yes	343 (35.1%)	119 (34.4%)	224 (35.6%)		373 (38.2%)	163 (43.5%)	210 (34.9%)		356 (37.2%)	168 (40.7%)	188 (34.5%)	
Affect regarding COVID-19 (from 1 to 7; higher values correspond to higher affect regarding COVID-19)	4.3 (1.0)	4.0 (1.1)	4.5 (0.9)	<.001	4.1 (1.1)	3.9 (1.3)	4.2 (0.9)	<.001	4.3 (1.0)	4.0 (1.2)	4.5 (0.9)	<.001
Presumed severity of COVID-19 (from 1 to 7; higher values correspond to higher severity)	4.0 (1.5)	3.7 (1.6)	4.1 (1.4)	<.001	4.1 (1.6)	3.8 (1.7)	4.2 (1.5)	<.001	4.1 (1.6)	3.9 (1.7)	4.3 (1.4)	<.001
Willingness to accept reduction of annual household income (%)	3.3 (6.0)	0 (0.0)	5.1 (6.9)	<.001	2.9 (6.4)	0 (0.0)	4.6 (7.7)	<.001	4.3 (11.0)	0 (0.0)	7.5 (13.7)	<.001

Wave 8: n = 976 individuals; wave 16: 977 individuals; wave 38: 958 individuals, in total and stratified by willingness to accept reduction of annual household income (WTA)

### Regression analysis

Two part models (for wave 8, wave 16, and wave 38) are presented in Table 2. Average marginal effects estimated by multiple Two Part Regression Models showed that in all three waves the maximum accepted reduction of income was significantly associated with presumed severity of COVID-19: After controlling for various covariates, the maximum accepted income reduction in wave 8 (wave 16; wave 38) increased with higher presumed severity of COVID-19 by + 0.42%, 95% CI: + 0.14% to + 0.71% (wave 16: + 0.46%, 95% CI: + 0.20% to + 0.72%; wave 38: + 0.75%, 95% CI: + 0.30% to 1.21%) per score point (scale ranging from 1 to 7). In wave 8 and wave 16 the maximum accepted income reduction was significantly lower for women than for men by − 1.06%, 95% CI: − 1.72% to − 0.39% and − 1.15%, 95% CI: − 1.74% to − 0.56%, respectively. Furthermore, in wave 8 only, maximum accepted income reduction was negatively associated with older age groups (e.g., ≥65 years: − 2.12%, 95% CI: − 3.73% to − 0.51% compared to 18–29 years), and positively associated with presence of children under the age of 18 (+ 0.90%, 95% CI: + 0.10% to 1.69%) and migration background (+ 1.54%, 95% CI: + 0.42% to + 2.65%). In wave 16 only, maximum accepted income reduction was positively associated with living in a big city (+ 1.28%, 95% CI: + 0.25% to + 2.31%), and negatively associated with living alone (− 1.39%, 95% CI: − 2.30% to − 0.49%) and being married/in relationship (− 1.02%, 95% CI: − 1.93% to − 0.12%). In wave 38 only, maximum accepted income reduction was positively associated with living in a small city (+ 2.26%, 95% CI: + 0.19% to + 4.32%), and the number of COVID-19 cases per 100,000 population (+ 1.65%, 95% CI: + 0.34% to + 2.96%). In all three waves, the likelihood for accepting any reduction in annual household income (logit model) was significantly positively associated with higher affect regarding COVID-19 (wave 8: OR = 1.44, 95% CI: 1.23 to 1.69; wave 16: OR = 1.25, 95% CI: 1.07 to 1.46; wave 38: OR = 1.43, 95% CI: 1.22–1.66), although the predictive margins were not significantly different from zero. For further details, please see Table 2.

**Table 2:** Two-part models (1. Logit 2. GLM^a^)

Independent variables	Wave 8			Wave 16			Wave 38		
	Logit OR (SE)	GLM b (SE)	Predict. margin	Logit OR (SE)	GLM b (SE)	Predict. margin	Logit OR (SE)	GLM b (SE)	Predict. margin
Gender: Female (Ref.: Male)	0.75*	−0.23*	−1.06**	0.68**	−0.28**	−1.15***	0.75*	0.07	−0.19
	(0.11)	(0.10)	(0.34)	(0.10)	(0.10)	(0.30)	(0.11)	(0.14)	(0.63)
Age category: - 30 to 49 years (Ref.: 18 to 29 years)	0.71	−0.36*	−1.79*	0.71+	−0.14	− 0.74	0.66+	− 0.03	− 0.76
	(0.16)	(0.16)	(0.77)	(0.15)	(0.13)	(0.47)	(0.14)	(0.19)	(0.89)
- 50 to 64 years	0.73	−0.40*	−1.91*	0.66+	−0.08	−0.66	0.61*	0.31	0.60
	(0.17)	(0.17)	(0.80)	(0.15)	(0.20)	(0.65)	(0.14)	(0.23)	(1.21)
- 65 years and over	0.64	−0.43*	−2.12*	0.91	−0.16	− 0.58	0.48**	− 0.04	−1.29
	(0.17)	(0.18)	(0.82)	(0.24)	(0.17)	(0.58)	(0.13)	(0.27)	(1.14)
Children (under 18 years): Yes (Ref.: Absence of children under 18 years)	1.00	0.27*	0.90*	0.95	0.05	0.10	0.79	0.04	−0.24
	(0.18)	(0.11)	(0.41)	(0.17)	(0.12)	(0.39)	(0.14)	(0.16)	(0.76)
Education: General qualification for university entrance (Ref.: absence of qualification for university entrance)	1.51**	0.03	0.55	1.54**	−0.02	0.37	1.12	− 0.09	− 0.18
	(0.23)	(0.09)	(0.34)	(0.22)	(0.11)	(0.34)	(0.16)	(0.14)	(0.66)
Town size: - Medium sized town (20,001 – 100,000) (Ref.: municipality/small town (1–20,000))	0.89	−0.09	− 0.39	1.20	−0.26*	− 0.48	1.09	0.27	1.22
	(0.16)	(0.11)	(0.39)	(0.21)	(0.12)	(0.33)	(0.19)	(0.18)	(0.81)
- Small city (100,001 – 500,000)	1.10	0.19+	0.82+	0.89	0.16	0.32	1.34	0.39+	2.26*
	(0.23)	(0.11)	(0.50)	(0.18)	(0.16)	(0.50)	(0.27)	(0.20)	(1.06)
- Big city (> 500,000)	0.92	−0.05	−0.25	1.16	0.34*	1.28*	1.18	0.12	0.73
	(0.18)	(0.14)	(0.48)	(0.23)	(0.14)	(0.53)	(0.23)	(0.18)	(0.76)
Region: East Germany (Ref.: West Germany)	0.82	0.25	0.60	0.60*	−0.04	−0.60	0.71+	0.10	−0.16
	(0.18)	(0.16)	(0.59)	(0.13)	(0.16)	(0.51)	(0.13)	(0.19)	(0.88)
Cases/100,000 population: Above median (Ref.: below median)	1.52**	0.05	0.61+	0.94	−0.11	− 0.38	1.19	0.31*	1.65*
	(0.24)	(0.10)	(0.37)	(0.15)	(0.12)	(0.38)	(0.17)	(0.13)	(0.67)
Relationship/Marriage: Yes (Ref.: no partnership/marriage)	0.95	−0.08	− 0.32	0.89	− 0.30*	−1.02*	1.03	−0.20	− 0.83
	(0.19)	(0.14)	(0.52)	(0.17)	(0.12)	(0.46)	(0.21)	(0.18)	(0.92)
Living situation: At least 2 individuals in the same household (Ref.: living alone)	1.32	−0.08	0.02	1.67*	0.32*	1.39**	1.14	0.13	0.79
	(0.29)	(0.14)	(0.52)	(0.35)	(0.14)	(0.46)	(0.25)	(0.20)	(0.94)
Migration background: Yes (Ref.: no migration background)	0.85	0.52***	1.54**	0.98	0.28+	0.78	1.43+	0.05	0.84
	(0.24)	(0.15)	(0.57)	(0.20)	(0.16)	(0.50)	(0.27)	(0.16)	(0.78)
Self-employment: Yes (Ref.: not self-employed)	0.79	0.17	0.31	1.11	0.33+	1.19	1.36	0.40+	2.71+
	(0.18)	(0.13)	(0.55)	(0.29)	(0.17)	(0.73)	(0.33)	(0.22)	(1.56)
Chronic disease: Yes (Ref.: no chronic diseases)	0.99	−0.20*	−0.65+	0.68**	−0.05	−0.51	0.70*	−0.04	−0.77
	(0.16)	(0.09)	(0.36)	(0.10)	(0.11)	(0.35)	(0.11)	(0.15)	(0.71)
Affect: COVID-19 (higher values correspond to higher affect)	1.44***	−0.08	0.12	1.25**	−0.11	− 0.10	1.43***	− 0.22**	− 0.35
	(0.12)	(0.05)	(0.19)	(0.10)	(0.08)	(0.23)	(0.11)	(0.08)	(0.39)
Severity: COVID-19 (higher values correspond to higher severity)	1.15*	0.08*	0.42**	1.15*	0.12**	0.46***	1.13*	0.13**	0.75**
	(0.06)	(0.04)	(0.15)	(0.06)	(0.04)	(0.13)	(0.06)	(0.05)	(0.23)
Constant	0.14**	2.19***		0.47	1.66***		0.32*	1.97***	
	(0.09)	(0.41)		(0.24)	(0.48)		(0.16)	(0.51)	
Observations	976	976	976	977	977	977	958	958	958

Two-part models with willingness to accept reduction of annual household income (in %) as outcome measure; ^a^ Generalized linear model (GLM) with log link and gamma distribution; *OR* Odds ratio; robust standard errors (SE) in parentheses; *** *p* < 0.001, ** *p* < 0.01, * *p* < 0.05, + *p* < 0.10

In a first robustness check (see Additional file 1; Supplementary Table 1), it was additionally adjusted for personal COVID-19 infection and COVID-19 infection in personal environment (wave 8 and wave 16 for reasons of data availability). In sum, findings remained similar in terms of effect sizes and significance. In a second robustness check, income (see Additional file 1; Supplementary Table 2) was added to our main model (wave 16 and wave 38 for reasons of data availability). Again, findings remained comparable in terms of effect sizes and significance. In a third robustness check, OLS regressions were used (see Additional file 1; Supplementary Table 3). Moreover, in a fourth robustness check, interval regressions were used (see Additional file 1; Supplementary Table 4). Again, it should be noted that findings remained similar when OLS or interval regressions were applied.

## Discussion

Based on general population samples from three waves of an online survey, this study aimed at assessing the willingness to accept a reduction of annual household income in order to bear the economic cost associated with measures against the spread of SARS-CoV-2 in Germany. While the first survey wave was conducted only a couple of days after the first (near) lock-down restrictions were lifted (April 2020), the second wave took place when infection rates were rather low and only few restrictions applied (July 2020), whereas the third wave was conducted during the second (near) lock-down (March 2021). While in the first wave almost 65% of respondent were willing to give up income in order to bear the economic cost, this proportion decreased to 57% in the third wave. The likelihood for accepting any reduction of income was consistently positively associated with higher presumed severity and higher affect regarding COVID-19 in all three waves. The mean maximum percentage of income participants were willing to give up was around 3% in the first and second wave, and increased to over 4% in the third wave, again with presumed severity of COVID-19 being positively associated with this in all three waves.

Compared to the magnitude of economic recession due to the COVID-19 pandemic, the mean maximum percentage of income participants are willing to give up started rather low in April and July 2020, but seemed to increase in March 2021, possibly indicating that the impact of the COVID-19 pandemic on the economy has only been gradually realized by the general population, probably due to income stabilizer and economic stimulus packages. The decreased proportion of respondents who were willing to give up income in March 2021 might be due to an increased proportion who in fact had already experienced a loss of income caused by the pandemic in 2020 (e.g. due to unemployment or pay cuts). Not surprisingly, perceived severity of COVID-19 and affect regarding COVID-19 tend to increase the willingness to accept reductions in income, as these variables reflect the subjective assessment of the health hazard associated with the infection [15]. Interestingly, in contrast to the authors’ expectations, the study from Poland cited above identified no impact of fear of COVID-19 on the willingness to bear the economic costs, which the authors attributed to the low level of fear in their relatively young sample (52% were aged 23 or younger) [6].

Significant positive associations between the accepted percentage of income reduction and other explanatory variables such as male gender, younger age, having young children, migration background and the incidence of COVID-19 were not consistently found in all waves and require further investigation.

To the best of our knowledge, this is the first study assessing the willingness of the general population to bear the economic cost of measures against the spread of SARS-CoV-2 in terms of willingness to accept a reduction of annual household income. We applied a simple form of the willingness-to-pay elicitation approach (in terms of giving up income), a contingent valuation method for elicitation of stated preferences, which has been applied in the framework of cost-benefit analysis in health care to estimate the monetary value of health effects of interventions [10, 11, 16]. We did not elicit willingness-to-pay in terms of an amount of money in Euro but as share of income because we assumed that this corresponds better to possible mechanisms the economic cost might have to be borne by the population (e.g. lower salaries, higher taxes, higher contributions to social insurance, lower profits of private businesses, higher inflation). However, it is important to note that we did not conduct a cost-benefit analysis but rather analyzed the general population’s attitude towards bearing the economic burden through giving up income. Most importantly, the effectiveness of the measures aimed at preventing the spread of SARS-CoV-2 (in particular in terms of reduced morbidity and mortality) has been largely uncertain at the time of the surveys and thus difficult or impossible to assess by the participants. Interestingly, in the cited experimental study from Poland [6], participants’ willingness to bear the economic costs in terms of unemployment and inflation was not sensitive to risk/uncertainty nor the perceived effectiveness of lockdown measures. The authors concluded that the decisions made by their study participants primarily aimed at protecting sacred values (such as human life) and were therefore in line with lexicographic decision models (such as the sacred value protection model [17]) rather than compensatory decision models. For a recent and more sophisticated (theoretical) model for estimating the willingness to pay for defined morbidity and mortality risk reductions incorporating various relevant aspects during the COVID-19 pandemic, please refer to another article [18].

Although a pretest was conducted which showed that the outcome measures had a high face validity, more sophisticated techniques of measuring preferences are required to validate our findings. Among other things, contingent valuation methods have been criticized for giving respondents little incentives to truthfully state their preference [9, 19] and being insensitive to scale [20]. Furthermore, in the questionnaire we did not explicitly state that there are various potential mechanisms of real net income reduction besides pay cuts (e.g., higher taxes, social security contributions, inflation) which the respondents might have factored in or not, and which could potentially bias their response. While the samples were representative of the German population aged 18 to 74 years in terms of age, gender and federal state, factors associated with the participation in online panels might be biased, and further research is required to investigate individuals aged 75 years and older.

## Conclusions

This study assessed the general population’s willingness to accept a reduction of annual household income and associated factors in order to bear the economic cost of measures against the spread of SARS-CoV-2 in Germany. The results show that the majority of respondents were willing to sacrifice income, with the willingness to give up income being consistently associated with variables related the perceived health hazard caused by the infection. However, the proportion of individuals who were willing to give up income seems to have decreased somewhat throughout the pandemic.

## Supplementary Information

**Additional file 1: **Two-part models (1. Logit 2. GLM^1^) with willingness to accept reduction of annual household income (in %) as outcome measure - with COVID infections (personal and in personal environment) as additional covariates (**Table S1**); Two-part models (1. Logit 2. GLM^1^) with willingness to accept reduction of annual household income (in %) as outcome measure - with household net income as additional covariate (**Table S2**); OLS regressions with willingness to accept reduction of annual household income (in %) as outcome measure (**Table S3**); Interval regressions with willingness to accept reduction of annual household income (in %) as outcome measure (**Table S4**).

## Data Availability

Data are not publicly available due to ethical restrictions but interested parties may contact the authors for more information.

## Abbreviations

CI: Confidence interval
COSMO: COVID-19 Snapshot Monitoring
COVID-19: Coronavirus disease 2019
GDP: Gross domestic product
GLM: Generalized Linear Model
OLS: Ordinary Least Squares
OR: Odds Ratio
Ref: Reference category
SARS-CoV-2: Severe acute respiratory syndrome coronavirus type 2
SD: Standard deviation
SE: Standard error
WTA: Willingness to accept reduction of annual household income
