# Supplementary material for: Willingness to bear economic costs of measures against SARS-CoV-2 in Germany
Source: BMC Public Health. 2021 Sep 17;21:1698. doi: 10.1186/s12889-021-11734-4 (PMC8446178; doi:10.1186/s12889-021-11734-4)
Supplement: Supplementary file 1 — Additional file 1: Two-part models (1. Logit 2. GLM1) with willingness to accept reduction of annual household income (in %) as outcome measure - with COVID infections (personal and in personal environment) as additional covariates (Table S1); Two-part models (1. Logit 2. GLM1) with willingness to accept reduction of annual household income (in %) as outcome measure - with household net income as additional covariate (Table S2); OLS regressions with willingness to accept reduction of annual household income (in %) as outcome measure (Table S3); Interval regressions with willingness to accept reduction of annual household income (in %) as outcome measure (Table S4). [file 12889_2021_11734_MOESM1_ESM.docx]

**Supplementary Table 1**. Two-part models (1. Logit 2. GLM^1^) with willingness to accept reduction of annual household income (in %) as outcome measure – with COVID infections (personal and in personal environment) as additional covariates

|  | Wave 8 | | | Wave 16 | | |
| --- | --- | --- | --- | --- | --- | --- |
| Independent variables | Logit OR (SE) | GLM  b  (SE) | Predict. margin | Logit OR (SE) | GLM  b  (SE) | Predict. margin |
| Gender: Female (Ref.: Male) | 0.75+ | -0.24* | -1.06** | 0.69** | -0.28** | -1.14*** |
|  | (0.11) | (0.09) | (0.33) | (0.10) | (0.10) | (0.30) |
| Age category: - 30 to 49 years (Ref.: 18 to 29 years) | 0.78 | -0.29+ | -1.35+ | 0.76 | -0.09 | -0.52 |
|  | (0.18) | (0.17) | (0.76) | (0.16) | (0.12) | (0.42) |
| - 50 to 64 years | 0.81 | -0.30+ | -1.36+ | 0.70 | 0.02 | -0.28 |
|  | (0.20) | (0.18) | (0.80) | (0.16) | (0.20) | (0.62) |
| - 65 years and over | 0.73 | -0.32+ | -1.51+ | 0.99 | -0.09 | -0.29 |
|  | (0.20) | (0.19) | (0.83) | (0.26) | (0.15) | (0.52) |
| Children (under 18 years): Yes (Ref.: Absence of children under 18 years) | 0.92 | 0.22* | 0.64+ | 0.92 | -0.04 | -0.18 |
|  | (0.17) | (0.10) | (0.39) | (0.17) | (0.11) | (0.36) |
| Education: General qualification for university entrance (Ref.: absence of qualification for university entrance) | 1.47* | 0.06 | 0.58+ | 1.51** | -0.01 | 0.35 |
|  | (0.23) | (0.09) | (0.34) | (0.22) | (0.11) | (0.34) |
| Town size: - Medium sized town (20,001 – 100,000) (Ref.: municipality/small town (1-20,000)) | 0.90 | -0.05 | -0.27 | 1.18 | -0.24* | -0.46 |
|  | (0.17) | (0.11) | (0.38) | (0.21) | (0.11) | (0.32) |
| - Small city (100,001 – 500,000) | 1.14 | 0.21+ | 0.90+ | 0.88 | 0.16 | 0.31 |
|  | (0.24) | (0.12) | (0.50) | (0.18) | (0.15) | (0.48) |
| - Big city (> 500,000) | 0.93 | -0.01 | -0.12 | 1.16 | 0.29* | 1.06* |
|  | (0.18) | (0.15) | (0.49) | (0.23) | (0.13) | (0.48) |
| Region: East Germany (Ref.: West Germany) | 0.82 | 0.23 | 0.56 | 0.62* | -0.03 | -0.52 |
|  | (0.18) | (0.15) | (0.54) | (0.13) | (0.16) | (0.49) |
| Cases/100,000 population: Above median (Ref.: below median) | 1.45* | 0.05 | 0.54 | 0.93 | -0.11 | -0.37 |
|  | (0.23) | (0.10) | (0.36) | (0.15) | (0.12) | (0.37) |
| Relationship/Marriage: Yes (Ref.: no partnership/marriage) | 0.95 | -0.10 | -0.38 | 0.89 | -0.31** | -1.05* |
|  | (0.20) | (0.12) | (0.47) | (0.18) | (0.12) | (0.44) |
| Living situation: At least 2 individuals in the same household (Ref.: living alone) | 1.33 | -0.04 | 0.15 | 1.64* | 0.31* | 1.34** |
|  | (0.29) | (0.13) | (0.48) | (0.35) | (0.14) | (0.46) |
| Migration background: Yes (Ref.: no migration background) | 0.82 | 0.49** | 1.41* | 0.96 | 0.23 | 0.60 |
|  | (0.17) | (0.15) | (0.56) | (0.20) | (0.15) | (0.48) |
| Self-employment: Yes (Ref.: not self-employed) | 0.78 | 0.05 | -0.09 | 1.08 | 0.27 | 0.94 |
|  | (0.18) | (0.12) | (0.47) | (0.29) | (0.17) | (0.68) |
| Chronic disease: Yes (Ref.: no chronic diseases) | 0.96 | -0.17* | -0.61+ | 0.65** | -0.14 | -0.79* |
|  | (0.15) | (0.09) | (0.34) | (0.10) | (0.11) | (0.36) |
| Affect: COVID-19 (higher values correspond to higher affect) | 1.45*** | -0.05 | 0.20 | 1.23** | -0.10 | -0.08 |
|  | (0.12) | (0.05) | (0.19) | (0.10) | (0.07) | (0.22) |
| Severity: COVID-19 (higher values correspond to higher severity) | 1.14* | 0.05 | 0.30* | 1.15* | 0.10** | 0.40** |
|  | (0.07) | (0.04) | (0.14) | (0.06) | (0.04) | (0.12) |
| Personal COVID-19 infection: - Don’t know (Ref.: No) | 0.99 | -0.17 | -0.51 | 0.84 | -0.07 | -0.34 |
|  | (0.23) | (0.13) | (0.42) | (0.19) | (0.15) | (0.43) |
| - Yes† | 2.39 | 0.90*** | 6.55* | 2.17 | 0.63** | 3.67* |
|  | (1.73) | (0.25) | (2.67) | (1.45) | (0.20) | (1.53) |
| COVID-19 infection in personal environment: - Don’t know (Ref.: No) | 0.98 | 0.32 | 1.17 | 1.09 | 0.57* | 2.05+ |
|  | (0.32) | (0.25) | (1.14) | (0.32) | (0.24) | (1.16) |
| - Yes†† | 1.74* | 0.05 | 0.71 | 1.55* | 0.23* | 1.09** |
|  | (0.40) | (0.12) | (0.49) | (0.30) | (0.11) | (0.42) |
| Constant | 0.16** | 1.54*** |  | 0.45 | 1.60*** |  |
|  | (0.09) | (0.38) |  | (0.23) | (0.47) |  |
| Observations | 976 | 976 | 976 | 977 | 977 | 977 |

^1^ Generalized linear model (GLM) with log link and gamma distribution; OR = odds ratio; robust standard errors (SE) in parentheses; *** p<0.001, ** p<0.01, * p<0.05, + p<0.10;

†: including: yes, confirmed; yes, but unconfirmed; yes, recovered

††: including: there are unconfirmed cases; there are confirmed cases; there are individuals who have recovered; there are deceased individuals

Please note that wave 38 was not used due to reasons of data availability

**Supplementary Table 2**. Two-part models (1. Logit 2. GLM^1^) with willingness to accept reduction of annual household income (in %) as outcome measure – with household net income as additional covariate

|  | Wave 16 | | | Wave 38 | | |
| --- | --- | --- | --- | --- | --- | --- |
| Independent variables | Logit OR (SE) | GLM  b  (SE) | Predict. margin | Logit OR (SE) | GLM  b  (SE) | Predict. margin |
| Gender: Female (Ref.: Male) | 0.72* | -0.19* | -0.84** | 0.77+ | 0.06 | -0.16 |
|  | (0.11) | (0.09) | (0.29) | (0.12) | (0.14) | (0.64) |
| Age category: - 30 to 49 years (Ref.: 18 to 29 years) | 0.73 | -0.26+ | -1.14* | 0.68+ | 0.08 | -0.22 |
|  | (0.16) | (0.14) | (0.56) | (0.15) | (0.17) | (0.82) |
| - 50 to 64 years | 0.71 | -0.29+ | -1.26* | 0.59* | 0.32 | 0.61 |
|  | (0.17) | (0.17) | (0.62) | (0.15) | (0.23) | (1.18) |
| - 65 years and over | 0.91 | -0.27 | -0.99 | 0.43** | 0.01 | -1.18 |
|  | (0.25) | (0.18) | (0.67) | (0.12) | (0.25) | (1.04) |
| Children (under 18 years): Yes (Ref.: Absence of children under 18 years) | 0.95 | 0.03 | 0.03 | 0.73+ | 0.07 | -0.21 |
|  | (0.18) | (0.12) | (0.40) | (0.13) | (0.16) | (0.76) |
| Education: General qualification for university entrance (Ref.: absence of qualification for university entrance) | 1.44* | -0.07 | 0.15 | 1.01 | -0.11 | -0.46 |
|  | (0.23) | (0.09) | (0.31) | (0.16) | (0.15) | (0.68) |
| Town size: - Medium sized town (20,001 – 100,000) (Ref.: municipality/small town (1-20,000)) | 1.23 | -0.17 | -0.25 | 1.16 | 0.42* | 1.88* |
|  | (0.23) | (0.11) | (0.31) | (0.22) | (0.18) | (0.83) |
| - Small city (100,001 – 500,000) | 0.88 | 0.20 | 0.44 | 1.45+ | 0.49** | 2.74** |
|  | (0.19) | (0.15) | (0.49) | (0.32) | (0.19) | (1.02) |
| - Big city (> 500,000) | 1.16 | 0.33* | 1.19* | 1.13 | 0.28 | 1.19 |
|  | (0.24) | (0.13) | (0.49) | (0.24) | (0.18) | (0.74) |
| Region: East Germany (Ref.: West Germany) | 0.68+ | 0.02 | -0.30 | 0.67* | 0.13 | -0.11 |
|  | (0.13) | (0.13) | (0.41) | (0.13) | (0.20) | (0.91) |
| Cases/100,000 population: Above median (Ref.: below median) | 0.96 | 0.07 | 0.15 | 1.30+ | 0.34* | 1.89** |
|  | (0.14) | (0.10) | (0.31) | (0.19) | (0.14) | (0.72) |
| Relationship/Marriage: Yes (Ref.: no partnership/marriage) | 0.84 | -0.34** | -1.23* | 1.08 | -0.19 | -0.70 |
|  | (0.18) | (0.13) | (0.49) | (0.24) | (0.19) | (0.94) |
| Living situation: At least 2 individuals in the same household (Ref.: living alone) | 1.40 | 0.24+ | 1.01* | 1.05 | 0.22 | 1.03 |
|  | (0.33) | (0.14) | (0.46) | (0.25) | (0.22) | (1.01) |
| Migration background: Yes (Ref.: no migration background) | 1.15 | 0.23 | 0.79 | 1.51* | -0.07 | 0.38 |
|  | (0.26) | (0.15) | (0.50) | (0.30) | (0.16) | (0.76) |
| Self-employment: Yes (Ref.: not self-employed) | 1.52 | 0.28+ | 1.38* | 1.49 | 0.26 | 2.02 |
|  | (0.44) | (0.16) | (0.70) | (0.39) | (0.20) | (1.29) |
| Chronic disease: Yes (Ref.: no chronic diseases) | 0.68* | 0.06 | -0.21 | 0.66* | -0.10 | -1.12 |
|  | (0.11) | (0.10) | (0.32) | (0.11) | (0.16) | (0.72) |
| Affect: COVID-19 (higher values correspond to higher affect) | 1.28** | -0.11 | -0.07 | 1.39*** | -0.17* | -0.19 |
|  | (0.11) | (0.07) | (0.21) | (0.12) | (0.08) | (0.38) |
| Severity: COVID-19 (higher values correspond to higher severity) | 1.15* | 0.11** | 0.44*** | 1.18** | 0.13** | 0.81*** |
|  | (0.07) | (0.04) | (0.13) | (0.07) | (0.05) | (0.23) |
| Household net income: - 1,250 Euro to lower than 1,750 Euro (Ref.: lower than 1,250 Euro) | 1.13 | -0.22 | -0.45 | 1.58 | 0.30 | 2.21 |
|  | (0.32) | (0.21) | (0.65) | (0.48) | (0.28) | (1.43) |
| - 1,750 Euro to lower than 2,250 Euro | 1.57 | -0.37+ | -0.54 | 1.30 | 0.28 | 1.69 |
|  | (0.44) | (0.21) | (0.63) | (0.37) | (0.28) | (1.33) |
| - 2,250 Euro to lower than 3,000 Euro | 1.30 | -0.25 | -0.41 | 1.16 | 0.11 | 0.67 |
|  | (0.35) | (0.22) | (0.66) | (0.33) | (0.27) | (1.14) |
| - 3,000 Euro to lower than 4,000 Euro | 1.41 | -0.03 | 0.31 | 1.92* | -0.21 | 0.15 |
|  | (0.40) | (0.21) | (0.70) | (0.58) | (0.28) | (1.10) |
| 4,000 Euro to lower than 5,000 Euro | 2.21* | -0.24 | 0.06 | 1.37 | -0.09 | 0.16 |
|  | (0.73) | (0.22) | (0.72) | (0.45) | (0.32) | (1.27) |
| 5,000 Euro and above | 1.57 | 0.31 | 1.73 | 1.63 | -0.07 | 0.48 |
|  | (0.58) | (0.34) | (1.49) | (0.60) | (0.28) | (1.17) |
| Constant | 0.31** | 1.85*** |  | 0.28* | 1.50** |  |
|  | (0.16) | (0.45) |  | (0.16) | (0.54) |  |
| Observations | 893 | 893 | 893 | 876 | 876 | 876 |

^1^ Generalized linear model (GLM) with log link and gamma distribution; OR = odds ratio; robust standard errors (SE) in parentheses; *** p<0.001, ** p<0.01, * p<0.05, + p<0.10

**Supplementary Table 3**. OLS regressions with willingness to accept reduction of annual household income (in %) as outcome measure.

| Independent variables | Wave 8 | Wave 16 | Wave 38 |
| --- | --- | --- | --- |
| Gender: Female (Ref.: Male) | -1.03** | -1.10* | -0.40 |
|  | (0.39) | (0.45) | (0.73) |
| Age category: - 30 to 49 years (Ref.: 18 to 29 years) | -1.83* | -1.22+ | -0.52 |
|  | (0.80) | (0.67) | (0.89) |
| - 50 to 64 years | -2.06* | -1.62+ | 0.70 |
|  | (0.84) | (0.83) | (1.20) |
| - 65 years and over | -2.18* | -1.35 | -0.86 |
|  | (0.91) | (0.85) | (1.26) |
| Children (under 18 years): Yes (Ref.: Absence of children under 18 years) | 1.23* | 0.57 | -0.14 |
|  | (0.51) | (0.55) | (0.69) |
| Education: General qualification for university entrance (Ref.: absence of qualification for university entrance) | 0.55 | 0.05 | -0.20 |
|  | (0.39) | (0.49) | (0.77) |
| Town size: - Medium sized town (20,001 – 100,000) (Ref.: municipality/small town (1-20,000)) | -0.41 | -0.41 | 1.21 |
|  | (0.44) | (0.38) | (0.88) |
| - Small city (100,001 – 500,000) | 1.28* | 0.47 | 2.35+ |
|  | (0.59) | (0.59) | (1.21) |
| - Big city (> 500,000) | -0.37 | 1.75* | 0.82 |
|  | (0.58) | (0.77) | (0.88) |
| Region: East Germany (Ref.: West Germany) | 0.22 | -0.32 | -0.50 |
|  | (0.58) | (0.64) | (0.94) |
| Cases/100,000 population: Above median (Ref.: below median) | 0.64 | -0.29 | 1.38* |
|  | (0.41) | (0.53) | (0.70) |
| Relationship/Marriage: Yes (Ref.: no partnership/marriage) | -0.36 | -0.70 | -0.51 |
|  | (0.56) | (0.45) | (0.84) |
| Living situation: At least 2 individuals in the same household (Ref.: living alone) | 0.03 | 1.12* | 0.37 |
|  | (0.57) | (0.49) | (1.04) |
| Migration background: Yes (Ref.: no migration background) | 1.76* | 0.91 | 0.52 |
|  | (0.80) | (0.75) | (0.91) |
| Self-employment: Yes (Ref.: not self-employed) | 0.91 | 1.66 | 2.87+ |
|  | (0.72) | (1.06) | (1.72) |
| Chronic disease: Yes (Ref.: no chronic diseases) | -0.18 | -0.48 | -1.13 |
|  | (0.41) | (0.46) | (0.92) |
| Affect: COVID-19 (higher values correspond to higher affect) | 0.08 | -0.29 | -0.36 |
|  | (0.22) | (0.30) | (0.38) |
| Severity: COVID-19 (higher values correspond to higher severity) | 0.53** | 0.71* | 0.85** |
|  | (0.18) | (0.28) | (0.30) |
| Constant | 1.15 | 2.40 | 2.13 |
|  | (1.52) | (1.65) | (1.87) |
| R² | .07 | .06 | .03 |
| Observations | 976 | 977 | 958 |

Notes: Unstandardized beta coefficients are displayed. Robust standard errors in parentheses; *** p<0.001, ** p<0.01, * p<0.05, + p<0.10.

**Supplementary Table 4**. Interval regressions with willingness to accept reduction of annual household income (in %) as outcome measure.

| Independent variables | Wave 8 | Wave 16 | Wave 38 |
| --- | --- | --- | --- |
| Gender: Female (Ref.: Male) | -0.87** | -0.95** | -0.43 |
|  | (0.29) | (0.33) | (0.53) |
| Age category: - 30 to 49 years (Ref.: 18 to 29 years) | -1.36* | -0.93+ | -0.45 |
|  | (0.58) | (0.48) | (0.67) |
| - 50 to 64 years | -1.48* | -1.21* | 0.27 |
|  | (0.62) | (0.60) | (0.86) |
| - 65 years and over | -1.56* | -0.89 | -0.81 |
|  | (0.67) | (0.62) | (0.91) |
| Children (under 18 years): Yes (Ref.: Absence of children under 18 years) | 0.88* | 0.35 | -0.08 |
|  | (0.38) | (0.40) | (0.51) |
| Education: General qualification for university entrance (Ref.: absence of qualification for university entrance) | 0.55+ | 0.20 | -0.17 |
|  | (0.29) | (0.35) | (0.55) |
| Town size: - Medium sized town (20,001 – 100,000) (Ref.: municipality/small town (1-20,000)) | -0.37 | -0.24 | 0.96 |
|  | (0.33) | (0.29) | (0.65) |
| - Small city (100,001 – 500,000) | 0.95* | 0.36 | 1.82* |
|  | (0.44) | (0.43) | (0.88) |
| - Big city (> 500,000) | -0.31 | 1.29* | 0.74 |
|  | (0.42) | (0.55) | (0.64) |
| Region: East Germany (Ref.: West Germany) | 0.09 | -0.31 | -0.38 |
|  | (0.43) | (0.46) | (0.68) |
| Cases/100,000 population: Above median (Ref.: below median) | 0.52+ | -0.16 | 1.01* |
|  | (0.30) | (0.38) | (0.51) |
| Relationship/Marriage: Yes (Ref.: no partnership/marriage) | -0.41 | -0.56+ | -0.35 |
|  | (0.41) | (0.34) | (0.62) |
| Living situation: At least 2 individuals in the same household (Ref.: living alone) | 0.16 | 0.96** | 0.28 |
|  | (0.42) | (0.37) | (0.76) |
| Migration background: Yes (Ref.: no migration background) | 1.22* | 0.59 | 0.53 |
|  | (0.58) | (0.54) | (0.67) |
| Self-employment: Yes (Ref.: not self-employed) | 0.61 | 1.14 | 2.22+ |
|  | (0.54) | (0.77) | (1.23) |
| Chronic disease: Yes (Ref.: no chronic diseases) | -0.13 | -0.43 | -0.95 |
|  | (0.30) | (0.33) | (0.66) |
| Affect: COVID-19 (higher values correspond to higher affect) | 0.16 | -0.14 | -0.21 |
|  | (0.16) | (0.21) | (0.28) |
| Severity: COVID-19 (higher values correspond to higher severity) | 0.38** | 0.51** | 0.69** |
|  | (0.13) | (0.20) | (0.22) |
| Constant | 0.81 | 1.79 | 1.68 |
|  | (1.13) | (1.19) | (1.41) |
| Observations | 976 | 977 | 958 |

Notes: Interval regression coefficients are displayed. Robust standard errors in parentheses; *** p<0.001, ** p<0.01, * p<0.05, + p<0.10.
